# Of (only) mice and men

**DOI:** 10.1038/sj.bjc.6690759

**Published:** 1999-11

**Authors:** B Leyland-Jones, C K Grieshaber

**Affiliations:** Department of Oncology, McGill University, Montreal, Quebec, Canada; Drug Discovery and Development, Karmanos Cancer Institute, Detroit, MI, USA


*British Journal of Cancer* (1999) **81**(5), 753–755
© 1999 Cancer Research Campaign
Article no. bjoc.1999.0759

Editorial

### Of (only) mice and men

**B Leyland-Jones[1] and CK Grieshaber[2]**

[1]Department of Oncology, McGill University, Montreal, Quebec, Canada, [2]Drug Discovery and Development, Karmanos Cancer Institute, Detroit, MI, USA

> Perhaps it is better to be irresponsible and right than to be responsible and wrong.
> 'Only constant repetition will finally succeed in imprinting an idea on the memory of the crowd.

When I took my A-level examinations in 1966, I was taught that in order to excel in the essay-writing section, one should always begin with a quotation and ascribe it to Winston S Churchill. 'After all', my teacher would say, 'Churchill said and wrote so much that no one will ever know the difference!' Well, 33 years later, my style has not changed … yet at least the first quotation is genuine Churchill … Party Political Broadcast, London, 26 August 1950. Moreover, we are certainly dealing with a heated issue of responsibility/irresponsibility and right/wrong. Every physician, scientist and patient desires the same goal – to obtain initial clinical experience of a new therapy with a minimum amount of animal toxicology, at a sufficiently safe starting dose and requiring the minimal number of dose calations to establish the appropriate dose.

Choosing the extreme conservative side of this paradigm necessitates the sacrifice of hundreds of thousands of animals unnecessarily and subject large numbers of patients to being treated at doses which are sub-therapeutic (Collins et al, 1986; Collins et al, 1990; Penta et al, 1992; Ratain et al, 1993; Simon et al, 1997).

Choosing the most aggressive approach, we save animals, money and time in the developmental process, but have the potential of killing patients by introducing drugs at unsafe doses.

The vast majority of pharmaceutical/biotechnology companies at the present time introduce their cancer drugs after full toxicity evaluation in both rodent and non-rodent species. By doing so, few would argue that they are 'right' in terms of conservatism with the patients' starting dose, and certainly 'right' in presenting regulatory bodies with more than enough information to satisfy the rigors of taking the drug into the clinic. The burning issue, of course, is whether they would still be 'responsible' in introducing a drug into the clinic with significantly less toxicology.

In the same year that I took my A-level examinations, EJ Freireich published a seminal paper on the quantitative comparison of toxicity of anticancer agents in mouse, rat, hamster, dog, monkey and man in *Cancer Chemotherapy Reports* (Freireich et al, 1966). They noted 'these results support the conclusion that the experimental test systems (the relationship of LD10 in animals to MTD in man) used to evaluate the toxicities of potential anticancer drugs correlate remarkably closely with the results in man.' Since that time (33 years ago!), the literature is replete with studies, reviews and editorials in an attempt to define the minimal data set required to predict a safe LD10/MTD (maximum tolerated dose) projection (Freireich et al, 1966; Homan, 1972; Goldsmith et al, 1975; Penta et al, 1979; Rozencweig et al, 1981; Grieshaber and Marsoni, 1986; Penta et al, 1992; Arbuck et al, 1996; Dent and Eisenhauer, 1996). The paper by Newell et al in this issue (see pp. *xxx*) is not only the latest paper in this field, but also one of the most comprehensive in its database, review and discussion. It contains derived data from a compilation of numerous preclinical and clinical studies. Indeed, prior to its publication, the vast majority of people in the field knew that the Cancer Research Campaign (CRC) experience with preclinical toxicology appeared to provide a safe starting dose of phase I clinical trials of cytotoxic anticancer agents.

This manuscript ventures somewhat further in its attempt to equate qualitative toxicity between mice and humans. This is difficult since only acute effects to a few tissues are examined in animal studies while human trials include a more comprehensive evaluation. Nevertheless, the qualitative associations are present for certain classes of new drugs. This area of the manuscript might benefit from the addition or the inclusion of National Cancer Institute (NCI) or industry experiences with 'non-rodent' studies to be able to state whether they would or would not add pertinent information, particularly on qualitative toxicities. This would enable the question of 'compared to non-rodent' to be addressed rather than simply stating the non-rodent species is/was not required. However, speaking personally (and, in an editorial, one is permitted to express personal opinions), I care somewhat less about the qualitative toxicity relationship. The critical issue to all of us must surely be the minimal data set of animal toxicology required to safely introduce the drug at an appropriately 'high' level in patients.

The discussion of the Newell manuscript refers to the critical examples where initiation of phase I trials at one-tenth of the mouse MTD/LD10 would have exceeded the human MTD. Without repeating the body of the text, the authors point out appropriately that compounds subject to intracellular metabolic activation may well require species-specific or other careful testing in selecting the suitable phase I trials starting dose. However, the authors conclude that for *routine* testing, one-tenth of the mouse MTD/LD10 represents a safe phase I trial starting dose.

It is an obligation for the writer of any editorial to be objective and as such, I have sought hard to fault the conclusions of this seminal paper. However, as I have consulted my toxicology friends and colleagues, I am assured that opinions on full non-rodent toxicology have significantly changed over the past 20 years; moreover, I am reassured that our regulatory colleagues are taking the data in this manuscript extremely seriously. It must be

*Received 22 April 1999*
*Accepted 23 April 1999*

*Correspondence to:* B Leyland-Jones. E-mail: leylandj@med.mcgill.ca clarified that the Fool and Drug Administration (FDA) at the present time requires a second, non-rodent species in order to test the safety of the *estimated starting dose only* for clinical studies and not full dose-escalating non-rodent toxicology. Moreover, the second non-rodent species can, to the best of my understanding, be a rabbit or another appropriate experimental species rather than a dog (DeGeorge et al, 1998). Of course, such an approach would need a full discussion with the FDA beforehand. However, the vast majority of drugs entering the clinic significantly exceed the FDA's absolute requirements.

And so to the second quotation on 'repetition' (a bottle of malt scotch to the first colleague who correctly e-mails me with the author) and to the reflection upon the remarkable life of Brian Fox who passed away on 28 March 1999; I find it the most humbling honour to write the editorial for his last manuscript. Chuck Grieshaber, who helped me write this editorial, first had the privilege to meet Brian Fox almost 20 years ago during the initial debates over the design and expectation of preclinical toxicology studies for new anticancer agents. At the time, Dr Fox and Dr Tom Connors were playing major roles in the establishment of the CRC Phase I/II Committee with the purpose of advancing new antitumour agents to clinical study as rapidly as possible. For a few years prior, Drs Fox and Connors were proposing a process where drugs would transit the laboratory–clinic bridge as a two-way street. The basic tenet held that the ultimate test of the value of a new modality rests in human studies. Non-clinical and experimental studies should support the trial not paralyse it! Amen. This is a simple paradigm that went largely unnoticed, or at least unheeded, in the USA until translational research became the byword for contemporary drug development over the past half decade involving the new molecular technologies and target-oriented clinical trials. Brian was continually stating the case for 'minimal' toxicology studies. By this he meant essentially the studies reported in the manuscript.

The themes of minimal toxicology studies and advancing new agents to clinical studies as rapidly as possible were brought to international impact by Brian's Vice-Chairmanship and Chairmanship of the Screening and Pharmacology Group of EORTC between 1979 and 1988 and by Brian's membership of the NCI-EORTC Joint Steering Committee between 1980 and 1993. Regrettably, although preclinical and clinical development times have overall decreased, 'constant repetition' with regard to minimal toxicology studies has not yet succeeded in 'imprinting the idea on the memory of the crowd'. I believe that the time has come for everyone involved in anticancer drug development to re-evaluate their current practice of animal toxicology. As a first step, we need to ask ourselves why toxicology testing on new cytotoxic or cytostatic agents in the USA so often significantly exceeds the current FDA requirements. Could not the PhRMA or some other pharmaceutical industry organization meet with the regulatory authorities to define a minimal agreed toxicology set for cytotoxic drugs? Secondly, in terms of the UK (and some other European countries), the unusual thing is not that the regulations are different but rather that 'exemptions' to some of the guidelines may apply if the trial is sponsored by a physician rather than a company. The DDX in the UK is one example. Should now the exemption not become the rule? Thirdly, the International Conference on Harmonisation of Technical Requirements for Registration of Pharmaceuticals for Human Use (ICH), established in 1990, is a unique project that brings together the regulatory authorities of Europe, Japan and the US and experts from the pharmaceutical industry in the three regions to discuss scientific and technical aspects of product registration. 'The objective of such harmonisation is a more economical use of human, animal and material resources, and the elimination of unnecessary delay in the global development and availability of new medicines whilst maintaining safeguards on quality, safety and efficacy, and regulatory obligations to protect public health' (ICH website: http://www.ifpma.org/ich1.html). It is imperative that their Steering Committee meet at the earliest possible convenience to discuss the significance of this publication. Two final points:

1. Clinicians are now saving time in the drug development process by other strategies such as enrolling only one patient per level and adopting more aggressive, often pharmacokinetically guided, dose escalation schemes. This often happens to compensate for a conservatively low starting dose. However, Chuck and I actually believe that the phase I assessment methods now considered are directly the result of phase I physicians not receiving the predictions required for sufficiently high starting doses and safe escalation procedures from the current toxicology data. Hence, what are we truly gaining from the time delay, not to mention the number of dogs sacrificed, in the non-rodent animal testing?
2. Now, more than ever, in the light of the hormonal, biological, immunological and targeted moieties, in which biologically active dose is often unrelated to maximally tolerated dose, entirely new models of animal testing are mandated. There is no question that much laboratory work is essential to understand the pharmacology of these new modalities; however, toxicology studies designed to define safety should never be limiting in the translation of drugs to clinical trial. We know that this is what Brian would have wished for as a significant slice of his professional legacy.

Brian did not have one distinguished career … but at least three. Beyond his oncology expertise, he was a Past-President and active member of the British Lichen Society and a member of the prestigious Linnaen Society. His books, papers and reports will form 'a substantial contribution to the annals of natural history to be consulted by many who follow in his footsteps (Dr AT McGown, Christie CRC Research Centre, Paterson Institute for Cancer Research, University of Manchester, UK). Thirdly, Brian was an avid historian of the Christie Hospital and made a unique collection of the research papers of distinguished scientists and physicians together with detail of the medical advances associated with oncology in this area of the world. In a paper he published in 1998 on the history of radium in medicine in Manchester (Fox, 1998), he spoke of the introduction of new remedies to treat cancer in the early part of this century. He noted that 'like a modern ethics committee, the hospital doctors tried to strike a balance between the aspirations of a novel treatment, while, at the same time, protecting the interest of the patient'. The best eulogy that we, as a cancer community, could offer to Brian is to strike the appropriate balance between minimal toxicology testing for cytotoxic agents while safely advancing these into current pharmaceutical practice. Moreover, in bringing the newer 'non-cytotoxic' treatment modalities into oncology practice, let us not repeat the errors of the past but instead design innovative preclinical testing requiring a minimum of toxicology in order to advance these new modalities into the clinic as rapidly as possible.

*© 1999 Cancer Research Campaign*

### REFERENCES

Arbuck SG (1996) Workshop on phase I study design. *Ann Oncol* **7**: 567–573

Collins JM, Zaharko DS, Dedrick RL and Chabner BA (1986) Potential roles for preclinical pharmacology in phase I clinical trials. *Cancer Treat Rep* **70**: 73–80

Collins JM, Grieshaber CK and Chabner BA (1990) Pharmacologically guided phase I clinical trials based upon preclinical drug development. *J Natl Cancer Inst* **82**: 1321–1326

DeGeorge JJ, Ahn C-H, Andrews PA, Brower ME, Giorgio DW, Goheer ME, Lee-Ham DY, McGuinn WD, Schmidt W, Sun CJ and Tripathi SC (1998) Regulatory considerations for preclinical development of anticancer drugs. *Cancer Chemother Pharmacol* **41**: 173–185

Dent SF and Eisenhauer EA (1996) Phase I trial design: are new methodologies being put into practice? *Ann Oncol* **7**: 561–566

Fox BW (1998) The history of radium in medicine in Manchester. *Clin Oncol* **10**: 115–124

Freireich EJ, Gehan EA, Rall DP, Schmidt LH and Skipper HE (1966) Quantitative comparison of toxicity of anticancer agents in mouse, rat, hamster, dog, monkey and man. *Cancer Chemother Rep* **50**: 219–244

Goldsmith MA, Slavik M and Carter SK (1975) Quantitative prediction of drug toxicity in humans from toxicology in small and large animals. *Cancer Res* **35**: 1354–1364

Grieshaber CK and Marsoni S (1986) Relationship of preclinical toxicology to finding in early clinical trials. *Cancer Treat Rep* **70**: 65–72

Homan ER (1972) Quantitative relationships between toxic doses of antitumour chemotherapeutic agents in animals and man. *Cancer Chemother Rep Part* **3**: 13–19

Penta JS, Rozencweig M, Guarino AM and Muggia FM (1979) Mouse and large-animal toxicology studies of twelve antitumour agents: relevance to starting dose for phase I clinical trials. *Cancer Chemother Pharmacol* **3**: 97–101

Penta JS, Rosner GL and Trump DL (1992) Choice of starting dose and escalation for phase I studies of antitumour agents. *Cancer Chemother Pharmacol* **31**: 247–250

Ratain MJ, Mick R, Schilsky RL and Siegler M (1993) Statistical and ethical issues in the design and conduct of phase I and phase II clinical trials of new anticancer agents. *J Natl Cancer Inst* **85**: 1637–1643

Rozencweig M, VonHoff DD, Staquet MJ, Schein PS, Penta JS, Goldin A, Muggia FM, Freireich EJ and DeVite VT (1981) Animal toxicology for early clinical trials with anticancer agents. *Cancer Clin Trials* **4**: 21–28

Simon R, Freidlin B, Rubinstein L, Arbuck S, Collins J and Christain Mc (1997) Accelerated titration designs for phase I clinical trials in oncology. *J Natl Cancer Inst* **89**: 1138–1147

*© 1999 Cancer Research Campaign*

